# Neoadjuvant therapy with anlotinib in a 5-year-old child with advanced papillary thyroid carcinoma: a case report

**DOI:** 10.3389/fped.2026.1780708

**Published:** 2026-05-13

**Authors:** Yuan Gong, Hongjiang Ma, Mingyang Lu, Qiuyu Meng, Bin Liu, Tingting Yang, Haifeng Ma, Yanjun Su, Ruochuan Cheng

**Affiliations:** Department of Thyroid Surgery, The First Affiliated Hospital of Kunming Medical University, Kunming, China

**Keywords:** anlotinib, children, locally advanced thyroid carcinoma, neoadjuvant therapy, oncologic therapeutic regimen

## Abstract

**Background:**

Pediatric papillary thyroid carcinoma (PTC) is frequently diagnosed at a locally advanced stage. Although anlotinib, an oral multi-target tyrosine kinase inhibitor (mTKI), has been employed in the treatment of locally advanced thyroid cancer (LATC) in adults, experience regarding its use in children remains limited.

**Case presentation:**

A 5-year-old boy was diagnosed with LATC, characterized by extensive lymph node metastases, encasement of multiple cervical vascular structures, and distant pulmonary metastases, rendering the tumor unresectable. In accordance with a multidisciplinary team (MDT) recommendation and existing pediatric evidence, neoadjuvant therapy with oral anlotinib (8 mg/day, administered on a 2 weeks on/1 week off schedule per cycle) was initiated. No severe adverse drug reactions were observed during treatment. Following 3 cycles of neoadjuvant therapy, the tumor demonstrated sufficient regression to achieve resectability, and the patient subsequently underwent successful staged surgical resection. Permanent hypoparathyroidism was the only complication.

**Methods:**

The patient's medical and imaging records were retrospectively analyzed. Tumor response was assessed using standardized Response Evaluation Criteria in Solid Tumors (RECIST) version 1.1, and adverse events related to targeted therapy were graded according to the Common Terminology Criteria for Adverse Events (CTCAE) version 5.0.

**Conclusion:**

This report describes a pediatric case of LATC treated with anlotinib-based neoadjuvant therapy, which may serve as a reference for future neoadjuvant treatment approaches in children with LATC.

## 1 Introduction

Pediatric differentiated thyroid cancer (DTC) is extremely rare. The incidence of PTC in the global population aged 0−19 years is approximately 0.46 per 100,000 (1). Data from the Global Burden of Disease (GBD) 2021 database indicate that the incidence of thyroid cancer in children and adolescents has increased significantly from 1990 to 2021. Compared to adults, pediatric thyroid cancer often presents with larger primary tumors, higher local invasiveness, and a greater propensity for cervical lymph node and distant metastases, particularly in children aged ≤ 10 years (2). Thyroid cancer in children and adolescents is frequently diagnosed at a locally advanced stage. At diagnosis, 50%–80% of children and adolescents with thyroid cancer already exhibit multi-compartment cervical lymph node metastases, a rate substantially higher than that observed in adults (3). Surgical management of LATC is highly challenging, especially in patients with T4b-stage disease. Patients with T4b lesions face extremely high surgical risks, and achieving R0 resection is often difficult. When formulating surgical strategies for pediatric patients with T4b disease, both tumor eradication and organ function preservation must be considered (4, 5). Neoadjuvant therapy, which can downstage tumors and improve clinical outcomes, has been recommended in various guidelines and is widely applied in the treatment of LATC. This report describes a case of a 5-year-old child with LATC treated with anlotinib. The case is presented alongside a review of domestic and international literature on the management of LATC, aiming to provide reference for neoadjuvant treatment approaches in pediatric LATC.

## 2 Case presentation

### 2.1 Treatment prior to anlotinib

**Figure 1:**
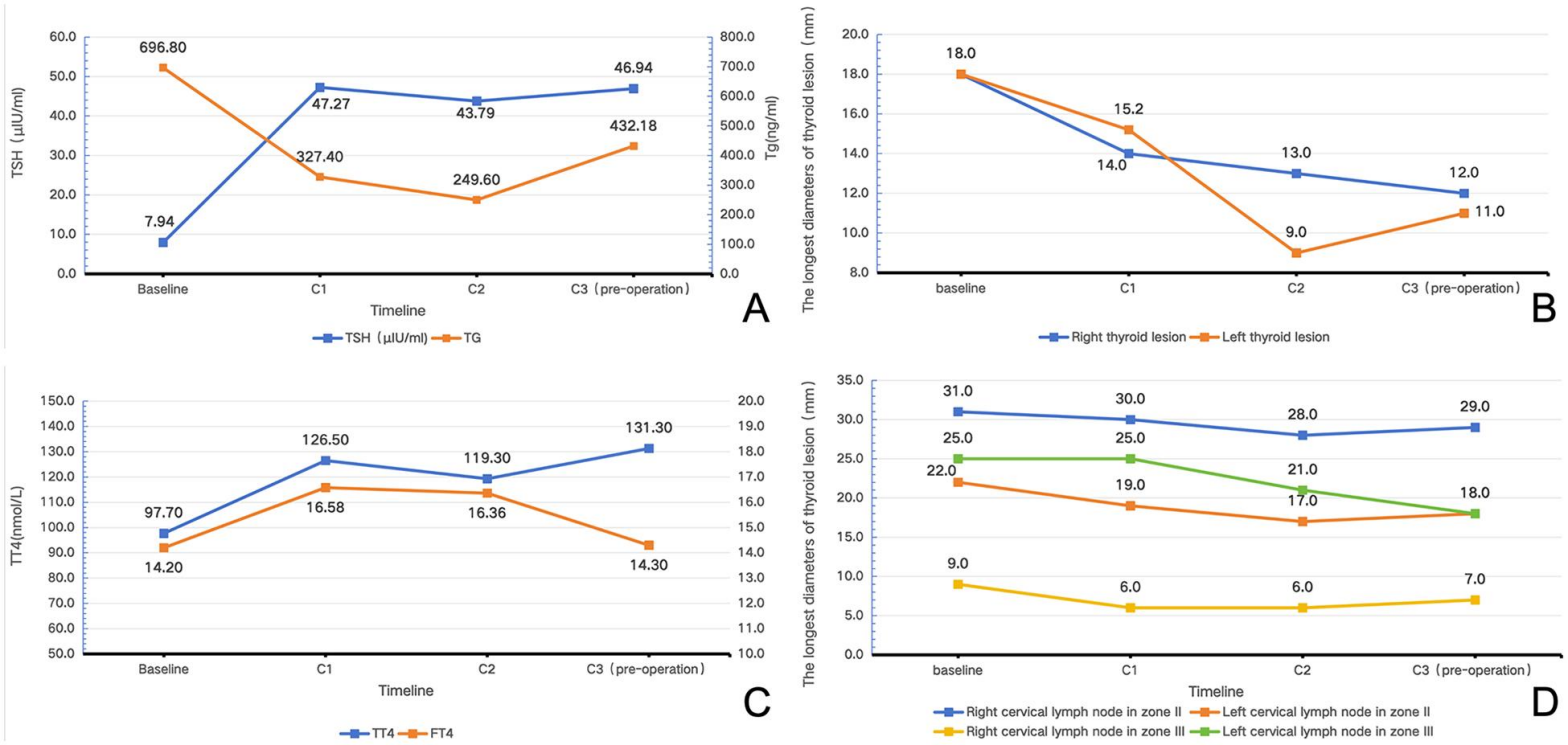
Changes in serum TSH, Tg, TT4, FT4 levels and the maximum diameter of tumors in various regions following anlotinib therapy. **(A)** Changes in serum TSH and Tg levels from baseline to C1,C2 and C3. (C1,C2,C3:refers to the cycles of anlotinib). **(B)** Changes of serum TT4 and FT4 levels from baseline to C3. **(C)** Changes in the longest diameter of left and right thyroid tumors from baseline to C3. **(D)** Changes in the longest diameter of left and right level II and level III lymph nodes from baseline to C3.

**Figure 2:**
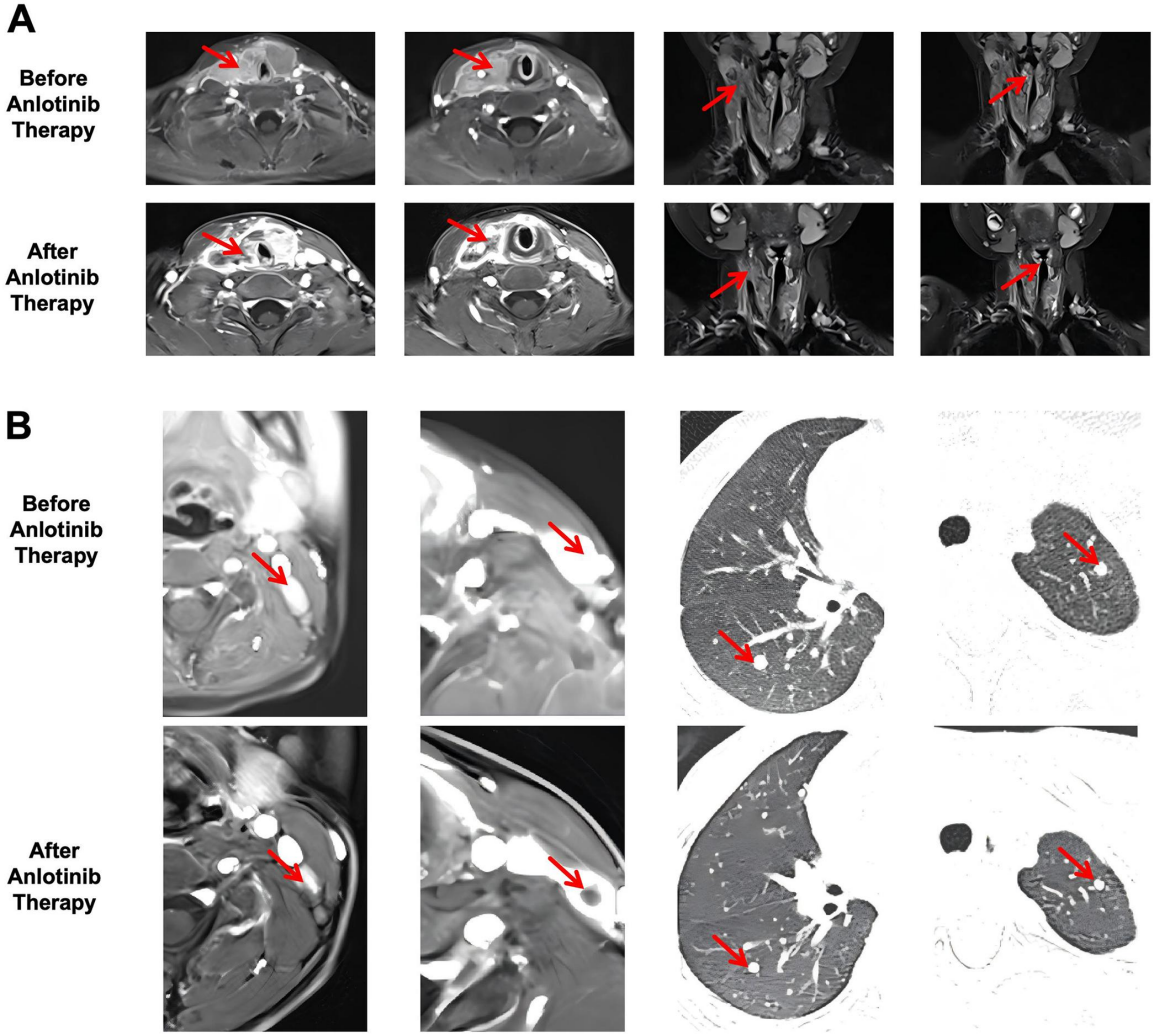
**(A)** imaging evaluation via MRI of the tumor's relationship with the right CCA, surrounding lesions, and tracheal compression before and after anlotinib treatment. From left to right: Thyroid tumor; Right CCA and surrounding lesions (sagittal view); Right CCA and surrounding lesions (coronal view); Tracheal compression. **(B)** Imaging evaluation of tumor size reduction via MRI and CT before and after anlotinib treatment. From left to right: Metastatic lymph node in left neck level II (MRI); Metastatic lymph node in left neck level IV (MRI); Metastatic lesion in right lung (CT); Metastatic lesion in left lung (CT). The key areas have been marked with red arrows.

A 5-year-old male child was admitted to our hospital due to “palpable neck mass for 3 months and hoarseness for 15 days.” 3 months earlier, an asymptomatic neck mass was noted. Subsequently, the family noticed hoarseness in the child and sought medical treatment at a local hospital. Due to limited experience in the management of pediatric thyroid disease at the local hospital, the patient was transferred to our hospital for further treatment. After admission, the physical examination revealed hoarseness, a full neck, and increased skin tension in the child. Nodules were palpable in both lobes of the thyroid gland, fused at the isthmus, measuring approximately 50  ×  50  ×  50 mm. The nodules were firm, irregular and immobile. A mass measuring about 40  ×  20 mm was palpable in the right level VI region, fused with the right thyroid tumor. Thyroid ultrasound indicated multiple solid hypoechoic nodules in both thyroid lobes, with the largest on the right measuring 18 × 15 mm and the largest on the left measuring 18 × 18 mm (Figure 1B-baseline). The mass in the right thyroid lobe was closely associated with the right common carotid artery (CCA) and internal jugular vein (IJV), with partial segments showing the right CCA and IJV traversing through the mass. Multiple lymph nodes with unclear corticomedullary differentiation were detected around sternocleidomastoid muscles, with the largest on the right measuring approximately 31 × 23 mm and the largest on the left measuring approximately 25 × 13 mm (Figure 1D-baseline). Thyroid magnetic resonance imaging (MRI) revealed diffuse enlargement of both thyroid lobes. Contrast enhanced computed tomography (CT) scan showed heterogeneous enhancement of the lesions, with bilateral involvement measuring approximately 55 × 29 × 48 mm. The right sided lesion partially encased the CCA, with adjacent tracheal compression. The narrowest diameter was approximately 3.54 mm (Figure 2A-before anlotinib therapy). Fine-needle aspiration and core needle biopsy confirmed PTC in bilateral thyroid nodules, and metastatic carcinoma (from the thyroid primary) was confirmed in bilateral cervical lymph nodes. No gene mutations were detected in BRAF^V600E^, TERT or NTRK. Fiberoptic laryngoscopic showed fixation of the right vocal cord. Chest CT scan showed diffuse micronodules in both lungs. The largest nodule in the right lung measured approximately 5 × 4 mm. The largest nodule in the left lung measured approximately 4 × 3 mm (Figure 2B-before anlotinib therapy). Thyroid function tests showed a thyroglobulin (Tg) level of 696.8 ng/mL and thyroid stimulating hormone (TSH) level of 7.94 *μ*IU/mL (Figure 1A-baseline), with no other significant abnormalities observed.

### 2.2 Multidisciplinary team assessment

Based on the child's post admission examinations, the clinical diagnosis is as follows: 1. Bilateral PTC (pT4bN1bM1, Stage II); 2. Secondary malignant tumor of bilateral cervical lymph nodes; 3. Secondary malignant tumor of both lungs; 4. Right vocal cord paralysis; 5. Subclinical hypothyroidism. Given that the primary tumor infiltrates the trachea, right recurrent laryngeal nerve (RLN), and encases the right CCA, the condition is classified as LATC. Due to the child's young age and the high surgical risk, the tumor is deemed unresectable. A MDT consultation has been requested, involving specialists from pediatric surgery, oncology, clinical pharmacy, critical care medicine among others.

The Department of Nuclear Medicine considered the child to have Radioiodine-Refractory (RAIR) DTC and recommended prioritizing surgical treatment, followed by radioactive iodine (RAI) therapy based on postoperative pathology results and residual lesions. However, the tumor was LATC, presenting a difficult airway and making safe and complete tumor resection unfeasible. Given the experience in neoadjuvant therapy for adult LATC, systemic neoadjuvant therapy was considered appropriate. Since no specific target gene mutations were detected in the genetic testing, and considering drug accessibility, anlotinib was selected as the targeted drug for neoadjuvant therapy under the guidance of clinical pharmacists.

### 2.3 Neoadjuvant therapy with anlotinib

After comprehensive consideration, the patient commenced oral anlotinib therapy. Lu et al. (6) previously conducted a clinical trial investigating anlotinib use in children, recommending a dosage of 8 mg/day for children weighing less than 35 kg, administered on a 2 weeks on/1 week off schedule per cycle. Based on existing pediatric evidence, the patient's body weight, and consultation with clinical pharmacists, a treatment regimen of 8 mg/day with a 2 weeks on/1 week off cycle was selected for this patient. After 2 cycles of anlotinib treatment, thyroid ultrasound demonstrated significant tumor reduction. The maximum diameter of the left lobe tumor decreased from 18 mm to 9 mm, and the right lobe tumor decreased from 18 mm to 13 mm (Figure 1B-C2). Bilateral cervical lymph nodes also showed marked reduction, with left level II cervical lymph nodes decreasing from 22 mm to 17 mm, and the largest right level II cervical lymph node decreasing from 31 mm to 28 mm. Ultrasound findings indicated therapeutic efficacy of anlotinib, and the patient was advised to continue treatment. Following completion of 3 cycles of anlotinib therapy, the patient's condition was reevaluated using CT and MRI. The thyroid tumor and right level II cervical lymph node were selected as target lesions, while lymph nodes in other cervical regions and pulmonary metastatic lesions were designated as non-target lesions. Results showed that compared to baseline, the thyroid tumor significantly reduced from 55 × 29 × 48 mm (length × width × height) to 43 × 16 × 50 mm, and the right level II cervical lymph node decreased from 31 × 23 mm (length × width) to 29 × 16 mm. Although RECIST 1.1 evaluation of anlotinib efficacy revealed that the sum of target lesion diameters decreased by approximately 24.3%, representing stable disease (SD) status, it is noteworthy that the thyroid tumor on MRI had markedly transformed into a hypovascular state (Figure 2A-after anlotinib therapy); pulmonary metastatic lesions showed reduction and disappearance (Figure 2B-after anlotinib therapy); and the narrowest intraluminal diameter of the trachea increased from 3.54 mm to 4.94 mm. Although tumor encasement of the right CCA showed no significant improvement, imaging revealed that lesions surrounding the right CCA had become markedly localized and hypovascular (Figure 2A-after anlotinib therapy). Serum Tg levels significantly decreased from 696.8 ng/mL at baseline to 249.6 ng/mL after C2 (Figure 1A-C2). Although a slight increase to 432.18 ng/mL was observed after the C3 (Figure 1A-C3), the level remained substantially below baseline (Tg antibody levels consistently remained within the normal reference range). The changes in TT4 and FT4 levels during the administration of anlotinib to the patient are shown in Figure 1C. During oral anlotinib administration, drug adverse reactions were monitored through regular hematological examinations and parental reports, with adverse events graded according to CTCAE 5.0. The patient experienced only grade 1 diarrhea, grade 1 alanine aminotransferase elevation, and grade 1 TSH elevation, demonstrating good tolerability. In summary, although anlotinib treatment did not achieve partial response (PR) according to RECIST 1.1 criteria, we consider that neoadjuvant therapy achieved clinically meaningful PR for this patient, creating a surgical opportunity. Ultimately, due to the patient's young age and inability to tolerate extensive surgery and anesthesia in a single procedure, staged surgical treatment was planned. The initial surgical procedure was scheduled as “total thyroidectomy (TT) + bilateral central neck dissection (CND) + right cervical lymph node dissection (LND),” followed by “left LND” after postoperative stabilization.

### 2.4 Surgery following anlotinib therapy

**Figure 3:**
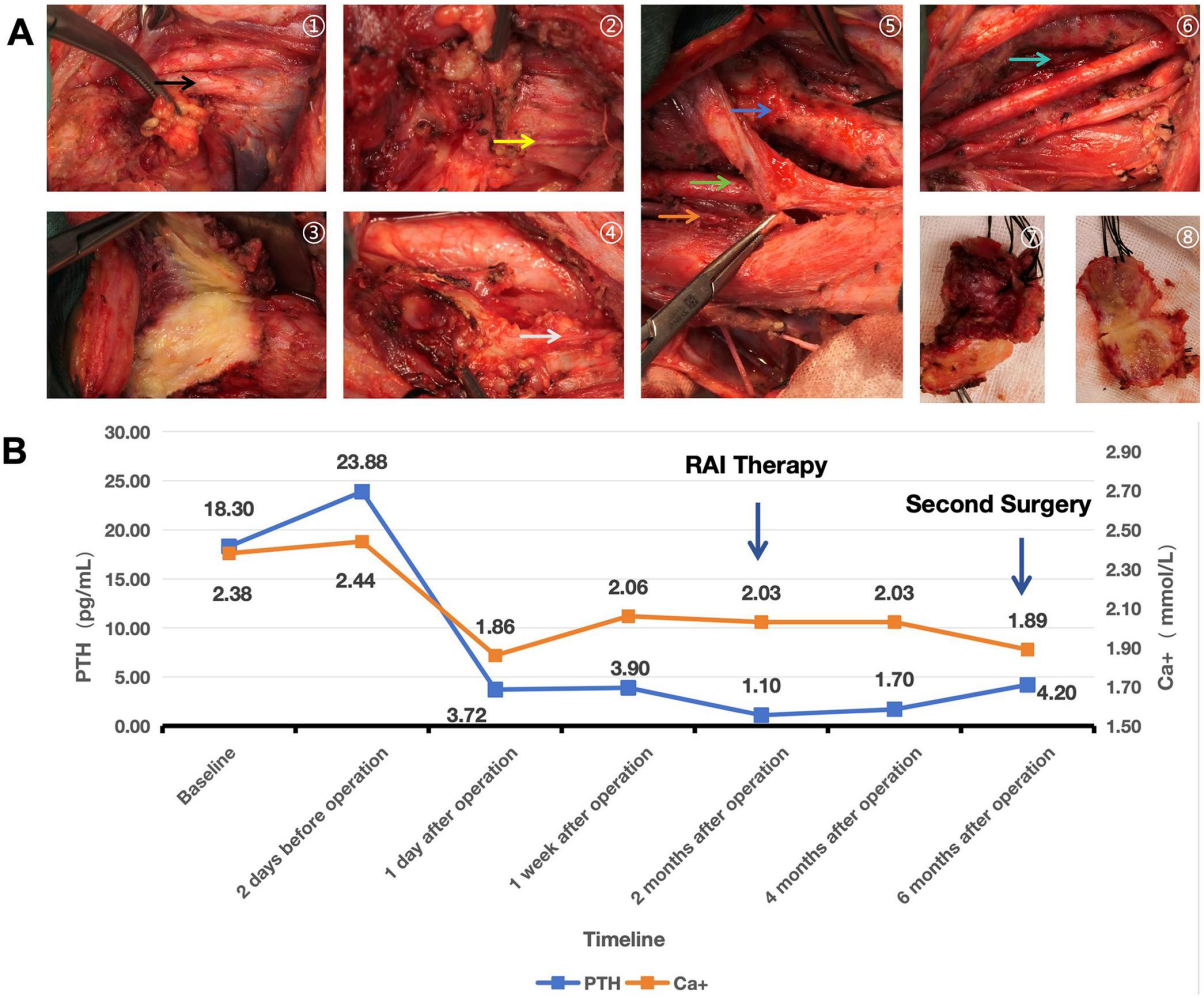
**(A)** intraoperative findings. Intraoperative findings during surgery. ①:Photo taken before incision was made. ②:Right thyroid tumor. ③:Tumor on the right tracheal wall.(apearing yellowish and chess-like due to tumor hypovascularity). ④:Left thyroid tumor.⑤-⑥:Photograph after tumor resection. ⑦-⑧:The appearance and profile of the rescted specimen. Vital structures are showed with arrows (Blue:Trach; Green:Right CCA; Orange:Rigth vagus nerve; Yellow:Right RLN; White:Left RLN; Black:Right IVJ; Cyan:Esophagus). **(B)** Changes in Serum PTH and Calcium Levels Following Surgical Treatment. Depicted are the levels from baseline to preoperatively at 2 days, and postoperatively at 1 day, 1 week, 2 months, 4 months and 6months.

Intraoperatively, the right thyroid tumor measuring approximately 30 × 20 mm and the left thyroid tumor measuring approximately 20 × 10 mm were observed. The tumors appeared yellowish and cheese-like in texture, suggesting a hypovascular state following anlotinib therapy. The surgical resection included TT, bilateral CND (with preservation of the left RLN), and right LND (with preservation of the right vagus nerve and right sternocleidomastoid muscle). Additionally, the involved segment of the right IJV, the right RLN were excised. The tumor was found to be invading the superficial layer of the first tracheal cartilage ring without entering the lumen. The tumor on the surface was sharply dissected while preserving tracheal integrity. Subsequently, the tumor on the surface of the esophagus was completely removed, and the muscularis layer was repaired with interrupted sutures. An R1 resection with no gross residual tumor was achieved. At the end of the procedure, reassessment revealed loss of signal in the left RLN, which was considered a temporary injury. No parathyroid glands were identified during the entire procedure. Routine dissection of the specimens showed no incidentally removed parathyroid glands in either the bilateral thyroid tissues or the bilateral central lymph nodes (Figure 3A). 6 months later, the patient underwent a second-stage left LND in our department.

### 2.5 Follow up and outcome

Following the initial surgical intervention, the pediatric patient received a single cycle of anlotinib therapy and was subsequently initiated on levothyroxine (L-T4) at a dosage of 62.5 μg/day for TSH suppression therapy. After 1 month of TSH suppression therapy, L-T4 administration was temporarily discontinued to facilitate a single session of RAI treatment. Post RAI therapy, L-T4 was reinstated and maintained at the original dosage. Concurrently, the patient received daily oral supplementation with calcium carbonate and calcitriol following the initial surgery. SPECT-CT imaging performed after RAI therapy revealed concentrated radioactive uptake in the left cervical level II-IV region, while no significant radioactive accumulation was observed in the right cervical lymph nodes, indicating the thoroughness of the initial surgical resection. Regular monitoring of serum parathyroid hormone (PTH) and calcium ion levels demonstrated that the patient remained persistent hypoparathyroidism and hypocalcemia following the initial surgery (Figure 3B), suggesting the development of permanent hypoparathyroidism. Thyroid function assessments conducted at 4 months and 6 months post-initial surgery (corresponding to 2 months and 4 months after completion of RAI therapy, respectively) revealed that serum Tg levels remained stable at approximately 35.0 ng/mL. Given that the residual tumor mass in the left cervical region had not been surgically excised, the biochemical efficacy of treatment could not be adequately evaluated. 6 months following the initial surgery, the patient underwent a successful second surgical intervention at our department. However, due to the patient's failure to return for scheduled postoperative follow-up visits, the relevant postoperative examination data following the second surgery are unavailable, precluding comprehensive evaluation of both structural and biochemical therapeutic outcomes.

## 3 Discussion

Surgical treatment, as the most critical step in the therapeutic cycle for children with LATC, should be prioritized (7). As clinicians, we must comprehensively evaluate the surgical benefits for LATC pediatric patients from multiple perspectives, including surgical difficulty, extent of resection, and potential surgical complications. Pediatric thyroid tumors often exhibit strong progression and invasiveness, which inherently translates to higher surgical complexity. For some LATC pediatric patients, tumors are frequently discovered at an advanced stage where R0 resection is unattainable, or even where surgical intervention is no longer feasible. Although neoadjuvant therapies can downstage tumors and create surgical opportunities for LATC pediatric patients, their administration may also induce severe fibrosis and tissue adhesion around the tumor. Several studies have demonstrated that following targeted drug therapy, peritumoral adhesive fibrous tissue significantly increases (8, 9), thereby elevating surgical difficulty and complication risks. The use of anlotinib is no exception. In this case, the patient developed significant tissue adhesions that were difficult to bluntly dissect after only 3 cycles of anlotinib treatment. Excessively high surgical difficulty often fails to provide direct surgical benefits to the patient; secondly, careful consideration must be given to the extent of resection and management of surgical complications. Yunushan et al. (10) indicates that when tumors exhibit high malignancy with lymph node and vascular invasion, surgical completeness must be emphasized, as patients with positive surgical margins may experience reduced overall postoperative survival. However, blindly expanding the surgical scope in pursuit of R0 resection and lower tumor recurrence risk may similarly lead to increased surgical complications, adversely affecting the patient's long-term quality of life. Therefore, LATC pediatric patients require thorough preoperative evaluation of surgical benefits by considering the aforementioned aspects to make the most beneficial decision for the child.

To systematically evaluate the current landscape of neoadjuvant therapy for pediatric LATC, we conducted a comprehensive search of the PubMed database using the following keywords: “Thyroid Neoplasms”, “Thyroid cancer”, “Thyroid carcinoma”, “Pediatric”, “Child”, “Adolescent”, “Childhood”, “Targeted therapy”, “Tyrosine kinase inhibitors”, “Lenvatinib”, “Sorafenib”, “Selpercatinib”, “Dabrafenib”, “Larotrectinib”, “Anlotinib”, and “Case Reports”. Following a rigorous screening process, 11 articles (comprising 8 case reports and 3 case series) were identified, reporting a total of 17 cases of pediatric neoadjuvant therapy. The therapeutic regimens included lenvatinib (*n* = 5) (8, 11, 12), sorafenib (*n* = 3) (13–15), selpercatinib (*n* = 5) (9, 16–18), and larotrectinib (*n* = 4) (19). Notably, no cases utilizing anlotinib for pediatric LATC were found. The summary content of the literature is shown in Table 1. The molecular profiles of the reported cases predominantly featured RET or NTRK gene mutations, a finding that is concordant with the established high prevalence of RET mutations in pediatric PTC (20). Selpercatinib and larotrectinib, functioning as highly selective TKI targeting RET and NTRK, have demonstrated satisfactory therapeutic efficacy. Beyond their direct anti-tumor effects, the capacity of selpercatinib and larotrectinib to induce re-differentiation in RAIR-DTC has been frequently documented. All 9 pediatric patients treated with these agents successfully transitioned from targeted therapy to RAI therapy during the treatment course (9, 16–19). While an early transition to RAI therapy may mitigate the adverse reactions associated with prolonged targeted drug exposure, whether this sequential approach effectively limits the progression of primary RAIR lesions necessitates longer-term follow-up for further elucidation. Conversely, lenvatinib and sorafenib, classified as mTKI, have also achieved significant therapeutic outcomes in pediatric LATC; however, their use appears to be accompanied by a higher risk of adverse events.

**Table 1:** Summary of literature reporting the use of targeted drug therapy in pediatric LATC patients. (**A**) Case reports. (**B**) Case series. (HFS: Hand-Foot Sydrome; NR: No Reports).

Author	Patients	Age	Clinical Pathological Characteristics	Drugs	Dose	Length of treatment	Adverse Effect (AE)	Outcomes
Julia R. Donner (11)	Female	7	PTC T4aN1bM1,stageII RET-PTC3 Fusion mutation	Lenvatinib	14 mg,qd(AE） →10 mg,qd→4 mg,qd(maintain)	>5.5years	HFS	Stable cervical lesions and pulmonary nodules
Dra. Noelia Dujovne (12)	Female	10	PTC T4aN1bM1,stageII RET-PTC3 Rearrangement	Lenvatinib	14 mg,qd→10 mg,qd(maintain)	NR	None	Stable cervical lesions and pulmonary nodules
Yousuke Higuchi (14)	Male	11	PTC T4aN1bM1,stageII No genetic mutations were found.	Sorafenib	200 mg,qd→400mg > 2years	2 years	Grade 1: HFS, Mild diarrhea, Elevated trarsaminases	Stable cervical lesions and pulmonary nodules
Steven G. Waguespack (13)	Female	11	PTC T4aN1bM1,stageII No genetic mutation result	Sorafenib	400 mg/d(AE) →200 mg/d（AE）→100 mg/d	9 months	Grade 3: Neutropenia Grade 2: Pruritic skin disease、Elevated lipase Grade 1: Thrombocytopenia, Hypocalcemia, Weight loss, HFS	Stable cervical lesions and pulmonary nodules
Taylor lnckie	Female	17	PTC T3aN1bM1,stageII NCOA4: RET Fusion	Selpercatinib	240 mg/d→4 months→operation→3 months 240 mg/d→RAI	7 months	Mild nausea	Marked regression of cervical and pulmonary lesions
Anna C. snydam	Female	13	PTC T1aN1bM1,stageII NCOA4: RET Fusion	Selpercatinib	240 mg/d	6 months	Grade 3: Weight gain	Significant reduction in pulmonary nodules
Harvey K. Chiu (17)	Female	10	PTC T4bN1bM1,stageII RET Recombination	Selpercatinib	240 mg/d→5months→RAI	>5 months	Grade 1: Intermittent rash	Stable cervical lesions and pulmonary nodules
Pallavi Iyer (15)	Male	8	PTC T4bN1bM1,stageII No genetic mutations were found	Sorafenib	Pre-operative: 400 mg/d Post-operation: 200 mg/d	3 months	Mild HFS	Stable cervical lesions and pulmonary nodules

Author	Number of Cases	Age	Drugs	Gene mutation type	Primary endpoint	Adverse event
Luz E. Castellanos (19)	4	6–16	Larorectinib	NTRK Fusion	Lung metastases shrank, enabling redifferentiation of RAIR lesions and restored iodine uptake	Grade 3: Hypocalcemia; Grade 2: Abnormal biochemical indicators; Grade 1: Arthralgia, Fatigue
Samara L. Potter (16)	2	16	Selpercatinib	NCOA4: RET Fusion	Cervical and pulmonary lesions remained stable without recurrence, with restored iodine uptake in the lesions	None
Priya Mahajan (8)	3	5–15	Lenvatinib	RET/NTRK Fusion	Stable cervical lesions and pulmonary nodules	Grade 3: Proteinuria, HFS

Anlotinib, as a mTKI, was initially designed to inhibit VEGFR2/3, FGFR1-4, PDGFR*α*/*β*, c-Kit, and Ret, thereby suppressing tumor angiogenesis and lymph node metastasis (21, 22). In recent years, several studies have suggested that VEGFR2 may be the primary target of anlotinib (23). By inhibiting the phosphorylation of VEGFR2, anlotinib can exert broader biological effects beyond anti-angiogenesis. Song et al. demonstrated that anlotinib not only inactivates the downstream PI3 K/AKT signaling pathway through VEGFR2 phosphorylation inhibition to exert anti-tumor activity, but also suppresses the AKT/HIF-1*α* pathway, reduces transferrin receptor (TFRC) expression, thereby attracting more CD8+ T cells into tumor tissues, modulating the tumor immune microenvironment, and enhancing the efficacy of anti-PD-1 therapy (24). Currently, although anlotinib has been listed as one of the optional mTKI in the expert consensus for the diagnosis and treatment of pediatric and adolescent DTC, and the objective response rate of anlotinib in adult LATC reaches 76.9% (25), reports on the use of anlotinib in special populations (such as children and elderly patients) for LATC treatment remain relatively limited. Su et al. (26) reported a 71-year-old elderly LATC patient successfully underwent surgical treatment after 4 cycles of anlotinib therapy without significant drug-related adverse reactions. Huang et al. and Zhao et al. indicated that anlotinib can provide significant survival benefits and safety in the treatment of elderly DTC and elderly MTC (27, 28). Lu et al. (6) conducted a clinical trial of anlotinib in 34 children with refractory tumors; although all patients reported adverse events, most were grade 1 or 2 in severity, with only 2 patients discontinuing anlotinib treatment due to adverse reactions. These research findings demonstrate the potential and value of anlotinib in the treatment of pediatric LATC.

The duration of anlotinib therapy and the timing of surgical intervention are critical determinants of the overall efficacy in pediatric patients with LATC (29). Currently, no unified standard exists regarding the optimal duration of anlotinib administration or the ideal window for surgical treatment in LATC. Although clinical studies on neoadjuvant therapy indicate a median progression-free survival (PFS) of 12.8 months for LATC patients treated with anlotinib (30), this does not imply that the disease can be indefinitely controlled by long-term medication. Prolonged exposure to anlotinib may not only precipitate severe adverse drug reactions but also induce fibrosis and scar-like changes within the tumor bed. These alterations can significantly increase surgical difficulty and compromise the surgical benefits for pediatric patients. Conversely, if surgical intervention is performed prematurely, the anatomical structures may remain indistinct and intimately associated with the tumor, thereby elevating the risk of injury to vital tissue structures. Consistent with 2 other patients treated with anlotinib reported by our center, all 3 cases demonstrated significant tumor shrinkage at C3 or C4 (26, 31). Whether C3 or C4 serves as an optimal time point to evaluate the efficacy of anlotinib requires confirmation through further clinical research.

Ensuring the long-term quality of life is equally important in the therapeutic management of pediatric LATC. Beyond monitoring the risk of tumor recurrence, implementing postoperative TSH suppression, targeted therapy, and RAI therapy, it is imperative to monitor and regulate growth-related indicators, bone metabolism markers, and hormone levels (32). Clearly, it is extremely challenging for a thyroid specialist to solely complete the comprehensive management of LATC patients. Therefore, in the diagnosis and treatment of pediatric LATC, a MDT is indispensable (33). Different disciplines can conduct individualized evaluations of LATC patients and formulate personalized postoperative treatment and follow-up plans, thereby improving the overall prognosis. Currently, the MDT model has become the standard paradigm for LATC treatment and is highly recommended in numerous studies and guidelines (34).

Anlotinib, as a mTKI, has demonstrated broad clinical applications across various malignancies. However, in the context of LATC with diverse molecular mutation profiles, the precision application of molecular targeted therapies warrants careful consideration by clinicians. Emerging evidence suggests that BRAF^V600E^ and TERT promoter mutations may serve as potential biomarkers for predicting therapeutic response to anlotinib (31). Beyond conventional genetic testing, tumor organoid models, specifically patient-derived organoids (PDOs), represent an innovative technological approach that can provide valuable guidance for individualized selection of targeted therapeutic agents. Xiang et al. (35) demonstrated that PDOs exhibit remarkable concordance with actual clinical outcomes in predicting the efficacy of radiotherapy and chemotherapy regimens. Notably, Zhu et al. (36) pioneered the application of PDOs technology for targeted drug selection in LATC patients. In their case report, PDOs were successfully established for a patient with thyroid cancer exhibiting extensive invasion of the entire larynx and trachea, revealing that anlotinib exerted superior inhibitory effects on the patient's cervical lesions. Following anlotinib administration, the patient's primary thyroid tumor demonstrated significant regression, ultimately enabling successful surgical intervention. The widespread adoption and implementation of PDOs technology across multiple medical centers should be actively promoted to enhance precision medicine approaches in thyroid cancer management.

## 4 Outcomes and limitations

We report a case of a pediatric LATC patient treated with anlotinib. In this case, anlotinib demonstrated favorable efficacy in the treatment of pediatric LATC without causing severe drug-related adverse reactions. This partially illustrates the value of anlotinib in the management of pediatric LATC. We hope this case can provide reference for neoadjuvant therapy in pediatric LATC patients. However, due to the relatively short follow-up duration, we are currently unable to evaluate the overall therapeutic efficacy in this patient. Conclusions regarding long-term outcomes still require extended follow-up observations. Furthermore, the therapeutic results of anlotinib in this pediatric LATC case may be coincidental, a single case report is insufficient to draw definitive conclusions regarding efficacy, and further clinical trials are needed to clarify the effectiveness of anlotinib in pediatric LATC. Finally, the indications, optimal dosing, and timing for surgical intervention in pediatric LATC patients receiving anlotinib therapy also require exploration through large-scale clinical studies.

## Data Availability

The datasets presented in this study can be found in online repositories. The names of the repository/repositories and accession number(s) can be found in the article/Supplementary Material.
